# Daily Newspaper View of Dengue Fever Epidemic, Athens, Greece, 1927–1931

**DOI:** 10.3201/eid1801.110191

**Published:** 2012-01

**Authors:** Christos Louis

**Affiliations:** Foundation for Research and Technology–Hellas, Heraklion, Greece; and University of Crete, Heraklion

**Keywords:** Aedes aegypti, dengue virus, dengue hemorrhagic fever, viruses, epidemic, vector-borne infections, newspaper, Greece

## Abstract

During the late summers of 1927 and 1928, a biphasic dengue epidemic affected the Athens, Greece, metropolitan area; >90% of the population became sick, and >1,000 persons (1,553 in the entire country) died. This epidemic was the most recent and most serious dengue fever epidemic in Europe. Review of all articles published by one of the most influential Greek daily newspapers (I Kathimerini) during the epidemic and the years that followed it did not shed light on the controversy about whether the high number of deaths resulted from dengue hemorrhagic fever after sequential infections with dengue virus types 1 and 2 or to a particularly virulent type 1 virus. Nevertheless, study of the old reports is crucial considering the relatively recent introduction of *Aedes albopictus* mosquitoes and the frequent warnings of a possible reemergence of dengue fever in Europe.

## Abstract 2

Controversy remains about possible dengue hemorrhagic fever during the epidemic.

The dengue fever (DF) epidemic of 1927–1928 in Greece, which first affected the Athens area, was the most recent dengue epidemic in Europe. Transmitted by *Aedes aegypti* mosquitoes, the epidemic probably involved dengue virus type 1 (DENV-1) and type 2 (DENV-2) (*1**–**4*). The rapid economic and social development of the continent has since led to dramatically reduced habitats of the vector and, most likely, to its elimination and is the main reason the likelihood of a similar event is small. Yet, the recent invasion of Europe by *Ae. albopictus* mosquitoes (*5*) and the marked ecology of this mosquito make emergence of a new DF epidemic possible, even if the impact of such an event is not expected to be of the same dimension as the epidemic described here.

Greece in 1927 lagged behind other countries in development. For example, most roads in Athens were unpaved; electric service was intermittent; a citywide sewage system was nonexistent; and the potable water supply was rudimentary, often forcing residents to store water in containers. Moreover, the population of the metropolitan Athens area, like the remainder of the country, had increased markedly because of the exchange of populations between Turkey and Greece after the war between the 2 countries and the defeat of Greece in 1922 (*6*). The Hellenic National Statistical Agency reported the arrival to Greece of ≈1 million refugees, leading to a 23.68% increase of the population during 1920–1928. In the Athens metropolitan area, the increase was ≈68%, and most of the newcomers were destitute and lived in extremely poor housing conditions (*7*).

In addition to these problems, the country was deeply divided politically into royalists and liberals, a division initiated early in 1915 by the rift between King Konstantin I and Prime Minister E. Venizelos about whether Greece should enter World War I. This division, exacerbated by the defeat of Greece in the Asia Minor campaign of 1921–1922, contributed to the lack of political solutions for the country’s major problems (*8*).

During this period of hardship, the DF epidemic struck Greece. The epidemic was then often called the dengue pandemic because almost the entire population of Athens (population ≈600,000) was affected. On the basis of the current definition of pandemic, the term epidemic is used here to describe the events of the summers of 1927 and 1928.

By focusing exclusively on all 561 items published in 1 daily newspaper during the epidemic, this review compares reports from 83 years ago with today’s knowledge. I chose the fully digitized archives of the Athens newspaper Η Καθημερινή (I Kathimerini) (*9*). This newspaper was, and still is, one of the major newspapers in Greece (currently first in daily circulation). During the time of the DF epidemic, the newspaper held a steady antiliberal and proroyalist position at a time when the liberal party ruled Greece. This position is often indicated in commentaries about the government’s handling of the epidemic, which will only briefly be mentioned here. Unless specifically referenced, all sources of original statements listed below (translated into English and appearing in quotes) are based on the pieces published by I Kathimerini.

## 1927

I Kathimerini first mentioned DF on November 1, 1927, not as news but rather as a short joke about financial matters, followed several days later by an advertisement for “FLY FUME” (ΦΛΑΪ ΦΙΟΥΜ), which “...protects you from the awful Dengue Fever, because it eradicates immediately the mosquitoes, which transmit it....” While publishing more jokes during the next few weeks and further advertisements for FLY FUME, I Kathimerini did not mention the DF epidemic until August 1928, when an article reported that the epidemic during the fall of 1927 had resulted in 3,000 cases with 0 fatalities; a few days later, the number of ill persons was reported as 75,000. Moreover, the second article suggested that many of these DF cases were initially mistaken for the yearly recurrence of “3-day fever,” which was known to be transmitted by phlebotomine sandflies. In August 1928, citing an unnamed physician, I Kathimerini reported that the previous year’s epidemic had started in mid-August in a middle-class household in the center of Athens through a young woman in whom DF symptoms developed 2 days after she arrived from Alexandria, Egypt.

## 1928

On August 2, 1928, when the second epidemic had been ongoing for several days, I Kathimerini began publishing frequent articles mentioning DF. An article in early September mentioned that the first 3 DF cases in 1928 had appeared in mid-July in the center of Athens among construction workers. This article also criticized the medical authorities for not issuing directions and for not taking any palliative and prophylactic measures despite the fact that “whole families stay in bed” or “big factories are forced to stop work.” The first real medical facts were published several weeks later in late August.

The headline of a long article appearing on August 15 was “25,000 Athenians are burnt by DF” and continued “... science remains speechless in front of the mysterious disease.” It described the symptoms of DF and mentioned a common prescription (“one spoon per hour of Riviera Potion 250 gr, Pyramidon 1 gr, Aspirin 1 gr”). The article concluded that “all measures and drugs suggested by [medical] science are symptomatic and not therapeutic.” It also reported that “those [persons] injected with 606 and Bismuth did not get DF” (606 was the synonym for arsphenamine/salvarsan).

The Table indicates the numbers of cases in the DF epidemic according to figures published by I Kathimerini (no figures were published after August 30, 1928). The Athens city center was hardest hit. An article on August 22 reported 30 deaths on the previous day, the first time that I Kathimerini reported deaths resulting from the epidemic (Figure). On that same day, another article noted that the fatal cases resulted from a “[coexistent] different organ affection” [*sic*]. Isolation of the DF “microbe” was unsuccessful, leading to the opinion that the transmitting mosquito might be poisonous. The vector was identified in different articles as *Stegomyia fasciata* mosquitoes, an old name for *Ae. aegypti* mosquitoes.

**Table:** Accumulated number of dengue fever cases as reported in the newspaper I Kathimerini during the 1928 epidemic, Athens, Greece, August 1928*

Date of report	No. cases			Notes
	Athens	Metropolitan area†	Greece	
	3,000–75,000			Mentioned in articles in 1928
Aug 15	25,000			
Aug 19	43,000			
Aug 23	80,000			
Aug 25	100,000			
Aug 27	150,000			
Aug 29	433,000	649,000		Indicated as 75% of population
Aug 30	461,000	693,000		Indicated as 80% of population
			959,884	Government statistics cited by Mavrogordatos (*8*)

*Blank cells indicate no information. †Includes the neighboring city of Piraeus (population ≈250,000) and the remaining suburban area that had a small population in the 1920s.

**Figure:**
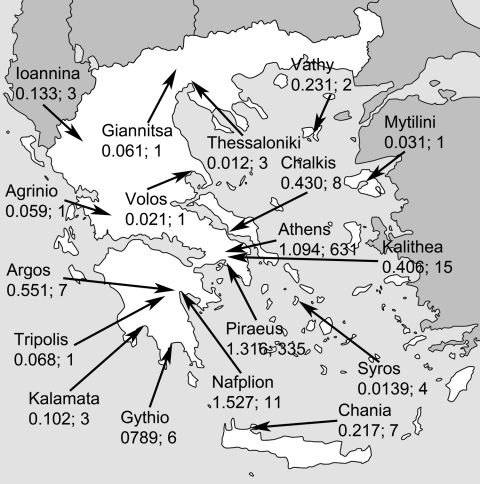
Map of Greece and deaths in the dengue fever epidemic, 1927–1928. The numbers are from the September 22, 1928, issue of the newspaper, I Kathimerini, and are ≈30%–40% lower than the official final death total. The first numbers indicate deaths per 1,000 inhabitants of each affected city (arrows); the number after the semicolon shows the total number of fatal cases from the epidemic in the respective location.

On August 24, some of the effects of the epidemic on society were reported for the first time. For example, the Athens–Piraeus Railroad used fewer trains, half the judges of the Athens Lower Court were absent, and the armed forces draft was suspended. Although more fatal cases were reported, hemorrhagic DF-like illness was not described, and deaths were attributed to older age or preexisting diseases. Some articles suggested that residents were afraid the epidemic might be yellow fever, but medical authorities explicitly denied this concern because “no patient ever showed any symptoms of hemorrhage” (the term σύμπτωμα in Greek covers both symptoms and signs). However, Cardamatis (*10*) described the course of the epidemic on the island of Aegina, located ≈25 km from the mainland, and stated that hemorrhage in all organ systems was the most common complication and cause of death. Furthermore, on August 25, I Kathimerini stated that “the present epidemic is malignant” and that neurologic disorders or cardiomyopathies often developed in the DF patients. To prevent complications, articles suggested that “intravenous salysalic [*sic*] acid, well sterilized [*sic*] should prevent cardiac diseases, especially myocarditis, ensuing from DF.”

Experts from Greece and abroad were asked for their views, but often the same expert cited in different articles in the same issue expressed contradictory opinions (e.g., possibility of DF transmission by house animals, excluded a few pages later). A common theme was that DF was associated with environmental degradation, and general measures for other diseases, such as cholera, should successfully curb the epidemic. The British power company performing public works and digging in Athens was accused of “providing breeding sites” for mosquitoes. Authorities seemed to be aware that only control of mosquitoes would help alleviate the epidemic and suggested that any “ditch should be covered as soon as possible and petroleum and/or lime should be used in order to prevent mosquitoes from breeding.” City authorities were advised to “wash the streets regularly and swamps should be dried the soonest possible.” These proposed measures were accompanied by proposals to “brush one’s teeth regularly” and take the drug “emostyrol.” Finally, several clergymen’s suggestion that “prayers and litanies would help end the epidemic” was made public.

On August 25, I Kathimerini reported the first cases outside the Athens area. Persons who had traveled to their birthplaces to vote in the general elections a few days earlier were held responsible for transporting the disease. On August 28, an article appeared that described what was then generally known about DF without citing the source of the information. Until that date, this article was 1 of only 2 scientifically sound articles describing the disease and its treatment. Additional articles contained amateur advice about how to cure DF (e.g., by “drinking milk and lemonade”). Development of an efficient drug also was reported (an “iodine solution per os” without further details), later followed by a letter from the chemist who developed it, cautioning that success achieved might have been purely coincidental.

More scientifically sound articles appeared in September. For example, the Minister of Health, an established pediatrician, explained how the “microbe” remained in Greece throughout the year; he had seen 1 DF case himself, 3 months before the new outbreak. He also explained that, after the acute phase, patients were expected to have lifelong immunity against dengue. Dr. Georges Blanc, head of the Hellenic Pasteur Institute, who was in France at the beginning of the epidemic, returned to Athens and granted an interview in which he tried to correct several statements. Moreover, a report signed by him and the health authorities also described facts about DF and its mode of transmission. I Kathimerini published a summary: the vector was *Stegomyia* mosquitoes, and the “microbe” had no relation to *Spirochaeta* (an association between DF and syphilis was considered possible by many physicians) or *Plasmodium laverani*.

One article cited a different newspaper claiming that the epidemic was not in fact DF but, rather, yellow fever. Two unnamed physicians asked for their opinion were cited as saying that if the disease were yellow fever, mortality rates would have been much higher. Initial reports on the epidemic in mid-August claimed that, compared with 1927, the new DF outbreak was much more malignant, on the basis of the lack of deaths reported during the previous year. I Kathimerini acknowledged the possibility that some deaths might have occurred in 1927 but, because of the previous ill health of the patients, could have been attributed to other causes.

The second month of the outbreak saw qualitatively different articles. Small news pieces about the everyday effects of the epidemic appeared frequently: entrance examinations to the universities were postponed, the army draft was deferred, theaters were closed, spouses and children were sent to resorts far from Athens, courts were closed because of the lack of judges and jurors, and the mode of taxation was changed to address lower income. Brief articles reported numbers of DF cases from Athens and elsewhere, including non-Greek cities such as Smyrna (now Ismir), Copenhagen, and Berlin, attributed to travelers from Greece. In mid-September, I Kathimerini reported that the epidemic was clearly weaker: “... the crowd at the horse races was again high after several weeks of low attendance.” On the same day, a 12-word item bore the headline, “ONLY TWO DEATHS!” On September 22, I Kathimerini published a report of the Ministries of National Economy and Health about the total number of deaths caused by the epidemic, aimed at calming foreigners wishing to travel to Greece. The numbers were corrected slightly a few days later, but the overall picture did not change (Figure). I Kathimerini did not subsequently publish new numbers of fatal cases.

The end of the epidemic saw a new kind of article about a potential reappearance in the following year. In a letter to I Kathimerini, a physician claimed that several patients bitten by infected mosquitoes late in the fall would become sick only in early spring. Therefore, in addition to mosquito control measures, an absolute isolation (i.e., quarantine) of the first patients of 1929 should prevent a new spread.

On October 29, I Kathimerini published a final report of the Nomiatros, the highest medical authority in the Νομός (i.e., county) of Attica; he claimed that DF could become endemic to Athens, but the fact that most persons living in the metropolitan area got sick and became immune, combined with the enhanced mosquito control measures that would be initiated, led him to conclude that DF would not be a real threat during the following years. That same day, I Kathimerini claimed that efficient measures against *Stegomyia* mosquitoes should suffice: covering any kind of water container and sewage-connected pipe or container and removing stagnant water from houses, kitchens, washing places, and flower pots.

## 1929–1931

Throughout the winter of 1928–29, sporadic articles citing DF, usually about unrelated matters, appeared in I Kathimerini. An often-recurring advertisement stated that many persons developed a hernia because of the “awful dengue disease,” and true relief from it could be found only in the “incomparable invention” of a Mr. Em. Rousos. Additionally, a short advertisement said, “Against Dengue, Life Savers [candies] triumphed; they act the same way against the flu.”

In May 1929, a series of articles in I Kathimerini described several findings and conclusions of investigations led by the Hellenic Pasteur Institute. In addition to confirmation of *Stegomyia* mosquitoes as the vector and lifelong immunity against DF, the scientists claimed that they also had produced an efficient vaccine; however, it was not to be used commercially. The vaccine had been tested, according to the newspaper, on members of the leper colony on the small island of Spinalonga and on patients of a public psychiatric hospital near Athens.

On July 7, 1929, I Kathimerini cited Ministry of Health officials as saying that no new DF case had appeared in Athens; only 2 days later, I Kathimerini reported that a person suspected to have DF was brought to the hospital. On July 21, three presumed DF cases were reported from Thessaloniki, the second largest city in Greece, ≈300 km north of Athens. Isolated cases of diseases resembling DF were reported sporadically throughout Greece but were never confirmed. Of those, the most important ones are ≈30 cases on the island of Syros in August 1931, of which a few supposedly were fatal. Yellow fever and an unknown spirochete infection were again discussed by the public (and discarded by health professionals); the unknown pathogen was declared to be transmitted by unidentified mosquitoes. To positively diagnose the disease, 3 psychiatric patients in Athens were inoculated with infected blood. One of the 3 who had not acquired DF 3 years earlier became sick with signs and symptoms of DF; the other 2, who had acquired DF during the epidemic, remained healthy. (Relatives of the first patient later sued the leader of the Hellenic Pasteur Institute team for attempted homicide. I Kathimerini did not report the outcome of this lawsuit.) Four months after these experiments, Institut Pasteur in Paris stated that the disease cases on Syros were caused by a spirochete.

## Discussion

Reading the old news items sparks 3 issues. First, did the high number of fatal cases in 1928 result from dengue hemorrhagic fever? Two reports support this notion on the basis of the limited serologic study of Athenians born around the time of the epidemic (*2**,**3*). The data are consistent with a sequential infection in some of the population with dengue viruses of different serotypes, a *conditio sine qua non* for the appearance of dengue hemorrhagic fever (here DENV-1 and DENV-2). This notion was contradicted by another serologic analysis that indicated only DENV-1 was involved in the epidemic (*11*). According to statements by health officials, the newspaper articles alleged that no hemorrhagic illnesses were observed. However, this information contradicts the findings from Aegina (*10*). Could the statement of the health officials have been false simply to calm the public?

Finally, several of the news items in 1928 cite physicians’ assertions that DF patients from the previous year never became re-infected. This fact does not exclude the possibility of an asymptomatic or a misdiagnosed first infection. The high death rate could have resulted from the incidence of 2 independent circumstances: highly virulent DENV-1 combined with the limited appearance of DENV-2, which could account for the hemorrhagic disease on Aegina and presumably elsewhere. The essence is that the question about the high death rate cannot be answered, and only a vast serologic study of the population, perhaps involving residents of the other areas in Greece where rates of death were high, might provide clues. However, 83 years after the epidemic, the pool of potential prospects continues to diminish.

The second issue regards the presence of *Ae. aegypti* mosquitoes in Greece. Except for infrequent sampling of specimens, the species seems to have disappeared from the country, as it has from most of the Mediterranean region (*12**,**13*). Two main reasons seem to account for its disappearance: 1) the widespread urban and rural antimalaria campaign based on insecticides and environmental management that lasted into the late 1950s (*14*); and 2) economic development that brought along measures and changes, such as running water, which helped reduce the number of containers in which mosquitoes reproduced. As in other places, malaria vectors, although found in much smaller numbers, have not disappeared from Greece and still can be identified in entomologic collections (*13*). As in other European countries, *Ae. albopictus* mosquitoes are now found in several areas in Greece (*15*; J. Vontas, pers. comm.).

The third issue is, of course, whether such a devastating epidemic could recur. The social conditions, including medical sciences and health care, now differ substantially from the late 1920s, and recurrence of an epidemic of such an extent and magnitude is unlikely in Greece or elsewhere in Europe. On the basis of present standards, in the absence of *Ae. aegypti* mosquitoes, even a major *Ae. albopictus*–transmitted DF epidemic is extremely unlikely, although minor events (in terms of numbers), such as the recent chikungunya epidemic in northern Italy (*16*), cannot be excluded. Recent cases of dengue (*17**,**18*) and chikungunya (*19*) in Europe resulting from international travel also support this notion (*4*).
